# Performance of the ChatGPT-5 Language Model in Solving a Specialty Examination in Balneology and Physical Medicine

**DOI:** 10.7759/cureus.96885

**Published:** 2025-11-15

**Authors:** Michalina Loson-Kawalec, Anna Kowalczyk, Dawid Szymanski, Patrycja Dadynska, Aleksander Tabor, Dawid Bartosik, Marta Zerek, Gracjan Sitarek, Bartosz Starzynski, Alina Keska, Bartlomiej Cwikla, Piotr Sawina, Tomasz Dolata, Adrianna Pielech, Maciej Majchrzak, Mateusz Podkanowicz

**Affiliations:** 1 Faculty of Medicine, University of Opole, Opole, POL; 2 Faculty of Medicine, University of Zielona Góra, Zielona Góra, POL; 3 Department of Internal Medicine, Karol Marcinkowski University Hospital, Zielona Góra, POL; 4 Department of Internal Medicine, Non-Public Health Care Institution (NZOZ) Hospital in Dzierżoniów, Dzierżoniów, POL; 5 Department of Internal Medicine, Wielospecjalistyczny Szpital Samodzielny Publiczny Zakład Opieki Zdrowotnej w Nowej Soli, Nowa Sól, POL; 6 Department of Internal Medicine, Lower Silesian Oncology Center, Wrocław, POL

**Keywords:** balneology, chatgpt-5, health technology, large language model (llm), physical medicine

## Abstract

Background

In recent years, there has been a breakthrough in the development of advanced computational systems based on neural networks. One such system is ChatGPT, first released in 2018, whose potential was quickly recognized, leading to its global popularity. Language models are increasingly capable of addressing complex problems, making them a promising tool to support the training of medical professionals. A particularly important aspect is AI’s ability to solve medical examinations, such as the Medical Final Examination (LEK) and the National Specialty Examination (PES), as well as international exams, including the United States Medical Licensing Examination and various specialty board examinations.

Objective

The objective of this study is to analyze the potential of the latest publicly available version of the ChatGPT-5 model in addressing examination questions in balneology and physical medicine as part of the PES. The study focuses on analyzing the accuracy of the model’s answers and evaluating the confidence of its decisions to assess its potential use as a supportive tool in medical education and specialty exam preparation.

Materials and methods

The experiment was based on the official Spring 2024 PES in Balneology and Physical Medicine, which consisted of 120 questions. The correctness of ChatGPT-5’s answers was verified against the official key prepared by the Center for Medical Examinations (CEM), while also recording the model’s self-declared confidence level on a 1-5 scale. Both the answer key and the examination database were obtained from the official CEM website. Prior to testing, ChatGPT-5 was introduced to the rules of the examination and provided with the full set of questions in Polish. The questions were divided into two groups: clinical and theoretical. Two questions were excluded due to inconsistency with current medical knowledge. Statistical analyses, including the chi-square test and the Mann-Whitney U test, were performed using Microsoft Excel (Microsoft Corporation, Redmond, WA, USA) and GraphPad Prism (GraphPad Software, San Diego, CA, USA).

Results

ChatGPT-5 provided 83 correct answers (70.34%), thereby surpassing the passing threshold. No statistically significant differences were observed between clinical and theoretical questions in terms of answer accuracy (p = 0.983), suggesting that the discrepancies were more likely attributable to random variation rather than true differences. Answer correctness was positively correlated with the model’s self-assessed confidence level (p = 0.029): the higher the declared confidence, the greater the likelihood of a correct response. The Mann-Whitney U test (p = 0.07) indicated that the difference in confidence levels between clinical and theoretical questions did not reach statistical significance (α = 0.05), although a trend toward potential differences was observed.

Conclusions

ChatGPT-5 demonstrated sufficient performance to pass the specialization examination in Balneology and Physical Medicine. The model displayed lower confidence in solving advanced clinical questions compared to theoretical ones. Answer accuracy was correlated with the assigned confidence level. While the Mann-Whitney U test (p = 0.07) did not confirm statistically significant differences in confidence between the two categories of questions, it suggested a possible trend. Further expert research is required before such models can be widely implemented in medical education.

## Introduction

ChatGPT, built on the GPT architecture, was launched in 2018 and rapidly gained widespread attention within both the scientific community and the general public. Its release was described as a milestone, as it markedly transformed the way researchers communicate and conduct scientific work. Within two years of its debut, the tool began to be applied to multiple aspects of academic activity, ranging from drafting and editing scientific texts, through literature searches, to the automation of code analysis. While this has contributed to increased productivity, concerns have also been raised regarding the quality and reliability of AI-generated content [1].

Modern language models are rooted in a seminal publication from 2017 [2], which proposed the use of attention as the core architectural principle. This laid the groundwork for successive generations of models (GPT-1, GPT-2, and GPT-3), each differing in scale and performance in natural language processing. Notably, GPT-3, released in 2020, with its 175 billion parameters, demonstrated the ability to perform tasks in both few-shot and zero-shot settings, enabling smoother human-machine interaction. GPT-4, announced in March 2023, further enhanced the architecture with multimodal capabilities, allowing image analysis and broadening practical applications, including in medicine [1,3].

Currently, GPT-5, officially released by OpenAI in August 2025, has replaced earlier versions such as GPT-4o and GPT-4.5 [1]. According to a study published by Emory University, GPT-5 demonstrated higher effectiveness in medical reasoning and multimodal diagnostics tasks than both the previous GPT-4o version and pre-licensed doctors. The model achieved a score of 95.84% on the MedQA test, representing an increase of 4.8 percentage points compared to GPT-4o. In tasks requiring the integration of patient history, medical images, and laboratory results, it reached 70% accuracy, which is nearly a 30-point improvement over its predecessor. Additionally, GPT-5 outperformed pre-licensed doctors by 24.23% in reasoning and by 29.40% in comprehension [4].

The development of AI has yielded tangible benefits in healthcare and biomedical sciences, including supporting faster diagnostic processes, enabling large-scale molecular data analysis, improving point-of-care diagnostic tools, and refining patient risk stratification. Clinical research published in high-impact journals, such as Tikhomirov et al. in 2024, confirms that AI improves diagnostic quality, particularly in radiology and other medical fields. However, the need for standardized research methodologies, multicenter analyses, and the inclusion of population-based data remains emphasized. Furthermore, the rapid implementation of AI in medical practice requires caution: it is essential to distinguish between experimental simulations and clinical validation while also considering cognitive factors influencing physicians’ decision-making [5].

The present study demonstrates that ChatGPT-5 is capable of passing the PES in Balneology and Physical Medicine, marking a significant advancement in handling complex medical questions. This example illustrates the rapid pace of AI development and the evolution of OpenAI’s models. Tracking AI progress requires systematic research documenting real-time changes.

The objective of this study was to evaluate the effectiveness of the ChatGPT-5 language model in answering National Specialty Examination (PES) questions in Balneology and Physical Medicine. Particular attention was paid to the accuracy of responses and the model’s self-assessed confidence. The analysis was based on a comparison of ChatGPT’s answers with the official answer key provided by the Center for Medical Examinations (CEM).

## Materials and methods

This study was conducted using GPT-5 on a single, randomly selected State Specialization Examination (PES) in Balneology and Physical Medicine (Spring 2024), obtained from the archives of the CEM in Łódź, Poland. The exam consisted of 120 multiple-choice questions, each with five options, only one of which was correct. Additionally, CEM excluded two questions as invalid, and their results were not included in the analysis. Appendix A presents the content of the questions translated into English along with the official CEM answers, GPT-5 chat answers, and the confidence level of the model’s answers.

The questions were divided into two categories: clinical questions, which consisted of patient-based scenarios requiring symptom analysis, interpretation of test results, and diagnostic or therapeutic decision-making; and theoretical questions, assessing general knowledge and familiarity with treatment standards.

Before the examination, the model was introduced to the rules, including the number of questions, response options, and the rule of one correct answer per question. Each answer generated by GPT-5 was compared with the official CEM key and recorded. After every response, the model was asked to rate its confidence on a 5-point scale: 1 - no confidence; 2 - low; 3 - moderate; 4 - high; and 5 - full confidence. All questions were provided in Polish to ensure alignment with the original exam materials.

Statistical analysis was conducted using Microsoft Excel (Microsoft Corporation, Redmond, WA, USA) and GraphPad Prism (GraphPad Software, San Diego, CA, USA). The chi-square test was applied to compare the distribution of correct and incorrect answers between categories, while the Mann-Whitney U test was used to assess differences in confidence levels between correct and incorrect responses. A p-value < 0.05 was considered statistically significant. Statistical analyses included the chi-square test and the Mann-Whitney U test, both performed in Microsoft Excel.

Because the investigators are located in different cities, collaboration between the research centers took place via online communication and document-sharing platforms, including Microsoft Teams (Microsoft Corporation), Facebook Messenger (Meta Platforms, Inc., Menlo Park, CA, USA), email, and Google Docs/Google Drive (Google, Inc., Mountain View, CA, USA). These tools supported real-time information exchange, collaborative document editing, and centralized storage of study materials. All manuscript sections prepared by different teams were peer-reviewed using the same platforms, ensuring consistency across contributions.

## Results

**Figure 1:**
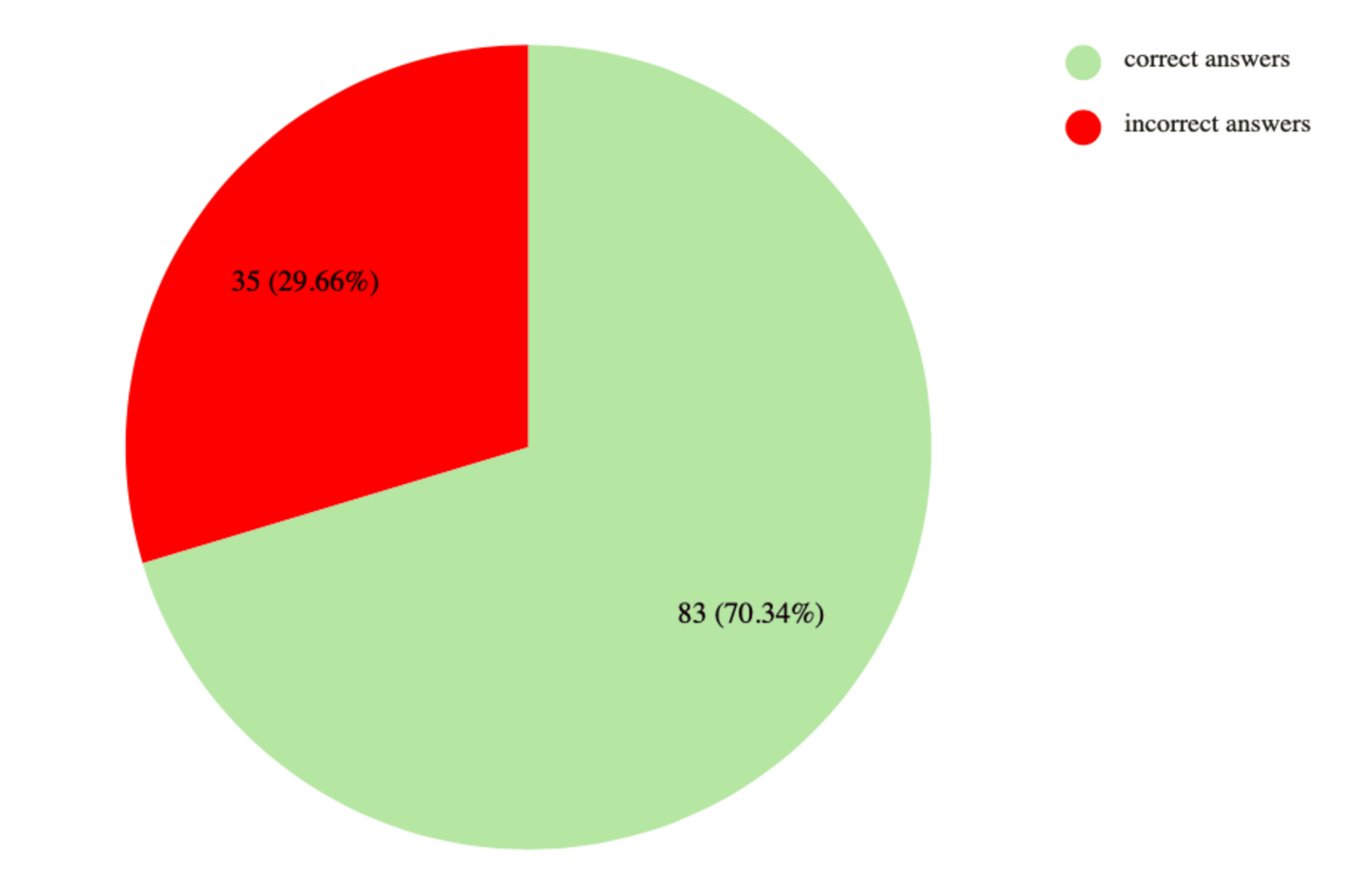
Accuracy of responses provided by ChatGPT-5 on the specialization exam in balneology and physical medicine (n = 118)

The ChatGPT-5 model was presented with 120 questions from the spring 2024 specialization exam in balneology and physical medicine (Table 1). The model answered 83 questions correctly (70.34%) and 35 incorrectly (29.66%) (Table 2, Figure 1). Two questions were invalidated by the Medical Education Center. No significant differences were observed in the performance on clinical versus theoretical questions (p = 0.98309; χ² = 0.00045) (Table 3). Confidence levels were found to correlate with answer accuracy (p = 0.02903; χ² = 4.76605) (Table 4). Moreover, the probability of obtaining a correct response was significantly higher when the model reported a higher confidence level (p = 0.0329) (Table 4). The Mann-Whitney U test result (p = 0.07137) suggests there is no statistically significant difference between clinical and theoretical questions.

**Table 1:** Comparison of ChatGPT-5 responses and reported confidence levels on the specialization exam in balneology and physical medicine The table presents the ChatGPT-5 model’s responses in relation to the correct answers provided in the answer key obtained from the CEM in Łódź. Each question includes the confidence level reported by the model, rated on a scale from 1 to 5 (1 - no confidence; 2 - low confidence; 3 - moderate confidence; 4 - high confidence; and 5 - complete confidence).

Question number	Official answer	ChatGPT response	Confidence level (1-5)
1	D	D	5
2	E	B	5
3	D	D	5
4	A	A	5
5	D	D	5
6	B	C	5
7	B	A	5
8	D	D	5
9	D	D	5
10	D	D	5
11	B	B	5
12	D	D	5
13	C	C	5
14	A	A	5
15	D	D	5
16	X	X	X
17	E	E	5
18	C	D	4
19	A	B	4
20	E	C	5
21	A	A	5
22	E	E	5
23	D	B	4
24	C	C	5
25	B	C	4
26	E	C	4
27	C	C	4
28	D	D	4
29	C	A	4
30	D	A	5
31	D	D	5
32	X	X	X
33	B	B	4
34	A	A	4
35	B	B	4
36	C	B	4
37	A	C	5
38	E	E	4
39	D	D	4
40	C	B	4
41	E	D	5
42	A	A	4
43	B	B	4
44	D	C	4
45	B	B	5
46	A	A	5
47	E	E	5
48	C	C	5
49	B	B	5
50	A	A	5
51	C	B	5
52	A	A	5
53	D	B	4
54	E	E	5
55	C	C	5
56	A	A	5
57	B	B	5
58	A	A	5
59	E	E	5
60	E	E	5
61	D	D	5
62	D	D	5
63	B	B	5
64	A	C	4
65	A	A	5
66	E	E	5
67	C	C	5
68	B	A	5
69	B	E	5
70	C	A	5
71	B	B	4
72	A	A	5
73	D	B	5
74	B	B	5
75	D	D	5
76	B	B	5
77	D	C	5
78	B	B	5
79	A	B	4
80	E	E	4
81	B	B	4
82	A	A	5
83	C	C	5
84	E	E	5
85	C	C	5
86	E	B	4
87	B	B	4
88	D	D	5
89	A	C	4
90	B	B	5
91	A	A	4
92	E	E	4
93	C	B	4
94	E	E	5
95	E	E	4
96	D	D	4
97	D	D	5
98	A	A	5
99	D	D	5
100	A	A	4
101	A	A	4
102	B	B	4
103	B	B	4
104	B	B	5
105	C	C	5
106	C	C	5
107	C	C	5
108	C	C	5
109	A	B	5
110	D	A	5
111	E	E	5
112	B	B	5
113	D	E	5
114	B	B	5
115	D	E	5
116	E	E	5
117	E	A	4
118	C	A	5
119	B	D	5
120	A	A	5

**Table 2:** Chi-square test assessing performance on clinical and theoretical questions ^*^ The full test contained 120 questions; two questions inconsistent with current medical knowledge were excluded from the analysis.

Question type	Correct response	Incorrect response	Total
Clinical	31	13	44
Theoretical	52	22	74
Total	83	35	118^*^
		p-value	0.98309
		χ²	0.00045

**Table 3:** Number of correct and incorrect responses by ChatGPT-5 on the specialization exam in balneology and physical medicine

Response type	Number of answers
Correct responses	83
Incorrect responses	35
Responses inconsistent with current knowledge	2
Total	120

**Table 4:** Chi-square test evaluating the association between confidence level and answer accuracy

Confidence level	Correct	Incorrect	Total
4	21	16	37
5	62	19	81
Total	83	35	118
		p-value	0.02903
		χ²	4.76605

**Figure 2:**
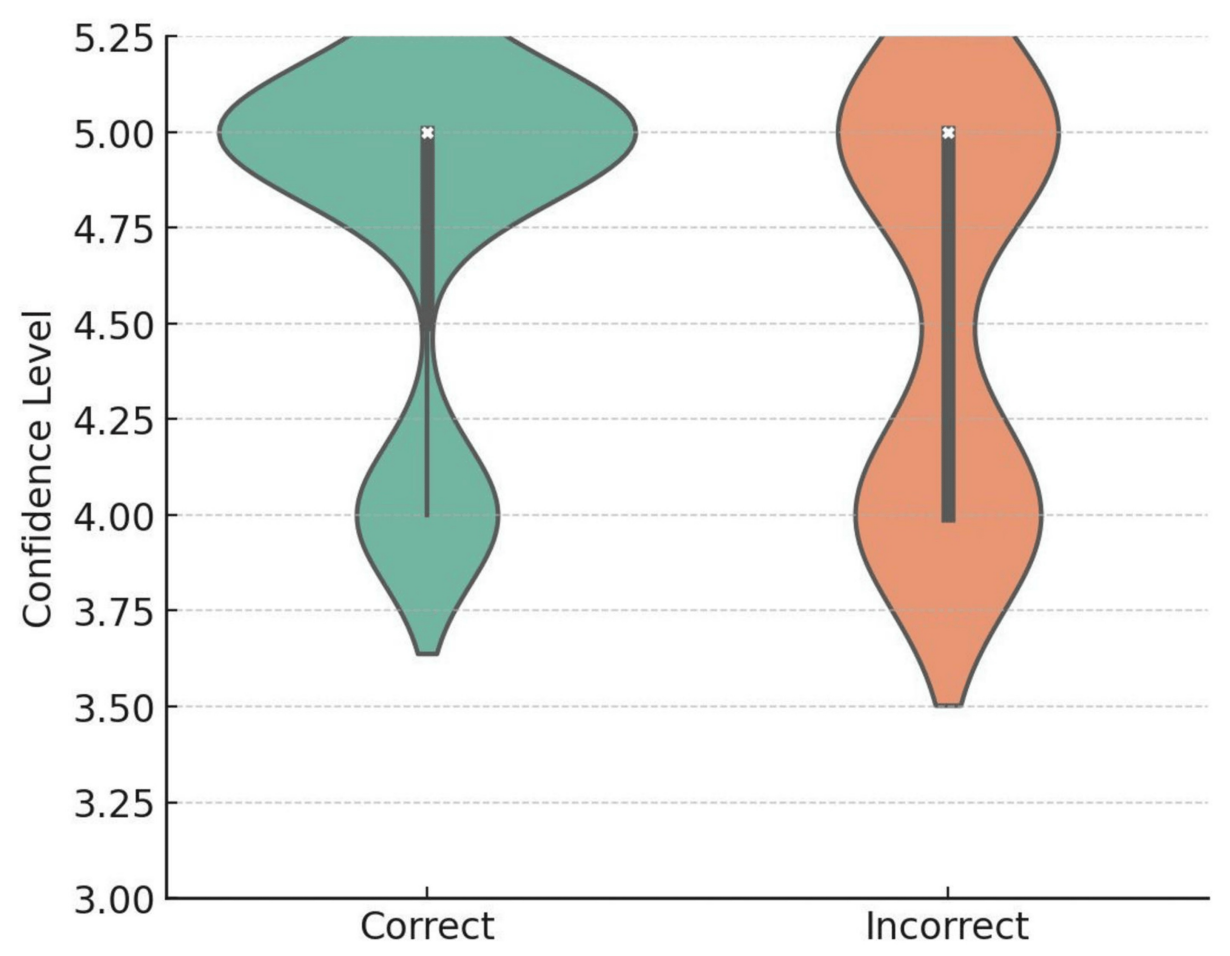
Reported confidence levels of ChatGPT-5 according to answer accuracy

Based on the analysis of the stated confidence levels in the responses provided by the ChatGPT-5 model, a significant difference was observed between correct and incorrect responses. A significant difference was identified when the confidence distribution was analyzed; correct responses were more likely to result in higher confidence levels, although incorrect responses were more likely to receive lower ratings (Figure 2).

## Discussion

PES in balneology and physical medicine represents a crucial stage in the process of obtaining specialization in this field. It requires candidates to demonstrate comprehensive theoretical knowledge and practical understanding of balneology and physical medicine, covering a wide range of conditions, diagnostic methods, and therapeutic procedures. The complexity and breadth of the topics make success in this exam a significant indicator of a specialist’s competence while also demanding integration of knowledge from various areas of medicine to support sound clinical decision-making.

This study evaluates the performance of the latest publicly available version of the ChatGPT-5 language model on examination tasks intended for future specialists in balneology and physical medicine. The model achieved a score of 70.34%, thereby meeting the passing threshold. Comparisons with earlier versions of OpenAI models in other medical domains demonstrate a clear trend of improving accuracy in addressing medical knowledge tasks.

AI is growing increasingly significant in the fields of physical medicine and balneology, where it aids in assessing the qualities of therapeutic mud and mineral water and helps to customize treatment [6]. Additionally, AI models enable the prediction of treatment results, therapeutic protocol optimization, and patient progress tracking, offering significant support for specialist training and clinical decision-making [7]. These technologies also offer remote patient state assessment and effective administration of balneological facilities [8]. Balneotherapy has been shown in prior research to be beneficial for rheumatic, dermatological, and mental health issues. AI integration can improve the accuracy of medical treatment and the efficacy of the educational process [6,7].

Hsieh et al. in 2024 demonstrated that the ChatGPT-3.5 version did not achieve the minimum score required to pass the Taiwanese Board of Plastic Surgery examination [9]. In a similar vein, a Polish study by Kufel et al. in 2023 on PES solving in radiology and imaging medicine revealed that ChatGPT-3.5 failed the examination [10]. In 2024, Yudovich et al. tested ChatGPT-3.5 and ChatGPT-4 on standardized urology knowledge assessments in the United States and demonstrated improved accuracy of responses with ChatGPT-4 compared to the earlier version. Nevertheless, neither model was able to meet the passing test requirements [11]. A study by Goodings et al. demonstrated ChatGPT-4’s promising potential in this field by demonstrating that it can manage the demands of complex medical examinations, especially when properly customized and taught in a specialized environment [12]. These findings, however, contrast with results from a study on the dermatology board examination [13]. Using 120 questions from the national specialty exam, the study assessed the performance of ChatGPT-3.5 and ChatGPT-4.0. In the Polish and English versions, ChatGPT-4.0 achieved minimum scores of 70% and 80%, respectively. Importantly, even in this narrowly focused specialty exam, the model demonstrated its potential as an educational tool, surpassing the passing threshold of 60% in both settings.

Our study’s findings emphasize ChatGPT-5’s adaptability and potential in medical education by showing that it performs similarly on clinical and theoretical questions (p = 0.983). A substantial association (p < 0.05) was found when the relationship between self-reported confidence and answer correctness was examined. Furthermore, the Mann-Whitney U test confirmed that correct answers had much greater confidence levels. When combined, these results suggest that confidence ratings could be a helpful assessment of how reliable model-generated responses are.

These observations align with previous studies on GPT-4, which demonstrated that higher confidence levels were associated with better quality responses and explanations. The literature also indicates that large language models effectively encode clinical knowledge [14] and perform well in solving educational and clinical tasks in both national and international settings [15,16]. In light of these findings, ChatGPT-5 may represent a valuable tool for supporting medical education and clinical decision-making, provided that its limitations are acknowledged and responses are subject to expert verification.

The low GPT-5 score in balneology and physical medicine (70.34%) may be related to the fact that these are niche fields with relatively little available data. The model relies primarily on publicly available sources, while practical knowledge and detailed procedures in these areas are rarely described in detail. Consequently, in tests requiring precise answers, it may perform worse than in better-documented medical fields.

The differences in performance achieved by subsequent versions of ChatGPT models across different medical disciplines suggest a need for further research into the use of AI in specialty testing. While ChatGPT-3.5 failed to pass exams in plastic surgery, radiology, and urology [9-11], newer versions, such as ChatGPT-4.0, achieved scores above the pass threshold in some areas, including dermatology [13] and nuclear medicine [17], as well as in our study using the ChatGPT-5 model. This variation suggests that the performance of AI models depends on both the software version and the specific exam material.

Further research is essential to better understand both the capabilities and limitations of AI across different fields of medicine. It is necessary to develop methods that enhance the accuracy of generated responses and to evaluate the extent to which AI models can support learning and preparation for specialty examinations. Systematic validation in diverse clinical contexts will enable the safe and effective use of AI in medical education and practice, while minimizing the risk of erroneous outputs that could lead to serious consequences.

The study also highlights directions for future research, including the validation of models on real patient data, support for integrating AI into the educational process, and the development of clinical decision support systems, which may contribute to improved diagnostics and therapy selection. Furthermore, the use of AI in medical education could reduce barriers and training costs, increasing access to high-quality educational tools regardless of location or institutional resources.

Our study has several significant limitations. First, the use of Polish questions in an analysis conducted in a tool primarily in English could have introduced translational distortions and affected the accuracy of the model’s responses. Linguistic differences and nuances in terminology could have hindered the interpretation of the question content and, to some extent, reduced the effectiveness of the obtained results. Another limitation is the narrow scope of the topics; the questions concerned only balneology and physical medicine, which limits the transferability of the findings to other fields of medicine. Furthermore, only responses from one model (ChatGPT-5) were analyzed, without comparison to other systems (e.g., GPT-4, Gemini, and Claude), which prevents us from assessing the relative effectiveness of different large language models.

Finally, confidence levels were assessed solely based on the model’s self-reported outputs, without independent validation in a clinical setting. Future studies should incorporate a broader range of questions, cross-model comparisons, and standardized translation procedures to enhance the reproducibility and reliability of results. Accordingly, AI should be regarded as a tool to support the learning process, rather than as a substitute for the expertise and experience of medical professionals.

## Conclusions

The study results indicate that reasoning-based models are capable of effectively analyzing complex cases in balneology and physical medicine and may, in the future, find practical applications in medical education and clinical practice, particularly in the context of technical and cost-related limitations. With the growing integration of AI into medical training, generative AI systems may enhance learning efficiency and preparation for specialization examinations.

Future research should focus on verifying the effectiveness of such models using real patient data, evaluating their impact on student performance, and developing strategies for integrating AI tools into both teaching and healthcare practice.

## Appendices

Appendix A 

**Table 5:** Questions and responses of the ChatGPT-5 model in the specialist examination in balneology and physical medicine The symbol "X" indicates a question invalidated by the Center for Medical Examinations.

Question	Official response	ChatGPT-5 response	Confidence Level
1. Which spa was founded by the renowned balneologist and politician Józef Dietl? A. Ciechocinek. B. Nałęczów. C. Karlovy Vary. D. Krynica. E. Marianskie Lazne.	D	D	5
2. Which type of climate is most appropriate for a patient with generalized osteoarthritis? A. maritime. B. lowland. C. foothill. D. mountain. E. any type.	E	B	5
3. Identify the foothill spas: A. Ciechocinek, Nałęczów, Swoszowice, Lądek-Zdrój. B. Cieplice Śląskie Zdrój, Świeradów-Zdrój, Gołdap, Świnoujście. C. Wieniec-Zdrój, Kudowa-Zdrój, Jedlina-Zdrój, Świeradów-Zdrój. D. Szczawno-Zdrój, Kudowa-Zdrój, Cieplice Śląskie Zdrój, Polańczyk. E. Horyniec-Zdrój, Wysowa-Zdrój, Rymanów-Zdrój, Gołdap.	D	D	5
4. The correct definition of a spa reaction is: A. the body's response to the cumulative effects of therapeutic stimuli applied serially, manifesting as a temporary exacerbation of symptoms and new subjective symptoms. B. the body's response to the cumulative effects of therapeutic stimuli applied serially, manifesting as a temporary exacerbation of symptoms and new subjective symptoms appearing in all patients during spa treatment. C. the body's response to a single therapeutic stimulus applied serially, manifesting as a temporary exacerbation of symptoms and new subjective symptoms. D. a set of disease symptoms occurring under the influence of a single stimulus during a therapeutic bath treatment. E. a set of disease symptoms occurring under the influence of the cumulative effects of electrical and electromagnetic stimuli.	A	A	5
5. The optimal temperature for a hydromassage in a thermal water bathtub is: A. 19°C. B. 21°C. C. 27°C. D. 37°C. E. 41°C.	D	D	5
6. The total time for one heating and cooling cycle in a Finnish sauna should not exceed: A. 5-7 minutes. B. 12-15 minutes. C. 20-30 minutes. D. 30-40 minutes. E. 45-55 minutes.	B	C	5
7. Indicate the true statement regarding the spa reaction: A. It is a very rare phenomenon, its pathogenesis is not fully understood, and it is an extreme increase in the body's reactivity during the ergotropic phase of the response to stimuli. B. It is a common phenomenon, occurring in full in 50% of patients, its pathogenesis is not fully understood, and it is an extreme increase in the body's reactivity during the ergotropic phase of the response to stimuli. C. It is a common phenomenon, occurring in full in 100% of patients, its pathogenesis is fully understood, and it is an extreme increase in the body's reactivity during the ergotropic phase of the response to stimuli. D. It is a common phenomenon, occurring in full in 50% of patients, its pathogenesis is not fully understood, and it is an extreme increase in the body's reactivity during the exotropic phase of the response to stimuli. E. The occurrence of a spa reaction is an indication for immediate discontinuation of the treatment.	B	A	5
8. Identify lowland spa towns: A. Wapienne, Długopole-Zdrój, Polańczyk, Cieplice, Czerniawa-Zdrój. B. Świeradów-Zdrój, Lądek-Zdrój, Krynica-Zdrój, Cieplice. C. Busko-Zdrój, Nałęczów, Ustroń, Duszniki-Zdrój. D. Gołdap, Wieniec-Zdrój, Supraśl, Nałęczów, Solec-Zdrój. E. Supraśl, Kudowa-Zdrój, Polanica-Zdrój, Rabka-Zdrój.	D	D	5
9. Identify the correct characteristics of the various skin phototypes: A. Skin type I - white skin, very high risk of burning, always tans. B. Skin type IV - olive (brown) skin, very high risk of burning, tans very poorly. C. Skin type V - brown (dark brown) skin, very high risk of burning, tans intensely. D. Skin type VI - black skin, never sunburns, always tans. E. All answers are correct.	D	D	5
10. The most important treatment modalities for stress urinary incontinence in women include: A. sodium sulfate and magnesium sulfate crenotherapy. B. four-chamber electro-water bath. C. multi-jet hydromassage, dry carbonic acid bath. D. physical therapy, kinesiotherapy. E. mud packs, dry ozone bath.	D	D	5
11 . Indicate the recommended balneophysiotherapy program for acne vulgaris: A. brine baths, partial peat packs, magnetic therapy. B. sulfur-hydrogen sulfur or ozone baths, peat paste packs, IR and UV radiation, radon baths. C. ozone baths, magnetic therapy, Sollux, galvanization. D. carbonic acid baths in water or gas, Bioptron, fango packs, IR radiation. E. none of the above.	B	B	5
12. Indicate the recommended treatment program for kidney stones at a spa: A. sulfur-hydrogen sulfur water crenotherapy, magnetotherapy, shockwave therapy. B. radon water crenotherapy, kinesitherapy, aquavibron. C. salt water crenotherapy, warm baths, and massage. D. hypoosmotic and hypothermal sorrel crenotherapy, mud packs, shortwave diathermy. E. mud packs, aquavibron, shockwave therapy, warm baths.	D	D	5
13. Indicate the recommended balneophysiotherapy program for diabetic polyneuropathy: A. sulfur-hydrogen sulfur baths, shortwave diathermy, fango. B. ozone baths, diadynamic currents, Sollux lamp, blue light. C. carbonic acid or vortex baths for the lower limbs, TENS, magnetotherapy, mud paste compresses. D. mechanical massage, douche, Finnish sauna. E. galvanization, brine baths, Sollux lamp.	C	C	5
14. Which treatment is most effective for sinusitis? A. Shortwave pulsed diathermy. B. diaelectric therapy. C. magnetotherapy. D. ultrasound. E. diadynamic current.	A	A	5
15. Specific water is low-mineralized or mineral water that contains at least: A. 4 mg of fluoride. B. 3 mg of iodide. C. 2000 mg of carbon dioxide. D. one specific ingredient with proven therapeutic effect. E. three specific ingredients with proven therapeutic effect.	D	D	5
16 . Select the true statements regarding contraindications to spa treatment: 1) malignant melanoma within one year of treatment completion, leukemia within one year of treatment completion, kidney cancer within five years of treatment completion; 2) compensated liver cirrhosis, lymphoma within five years of treatment completion, schizophrenia treated without symptoms for several years; 3) decompensated liver cirrhosis, stomach cancer within one year of treatment completion, unstable affective disorder; 4) paroxysmal atrial fibrillation without anticoagulant prophylaxis, myeloid leukemia more than five years after treatment completion, no indications for spa treatment; 5) chronic renal failure, uncontrolled diabetes, kidney cancer more than five years after treatment completion. The correct answer is: A. 2, 3. B. 3, 5. C. 3, 4. D. 1.5. E. all of the above.	X	X	X
17. What is the upper age limit for a patient who can be referred for spa treatment? A. 85 years old. B. 90 years old for women, 85 years old for men. C. 95 years old. D. 100 years old. E. There is no upper age limit.	E	E	5
18. A 58-year-old woman with scleroderma limited to the skin was admitted to a health resort. The patient had no known malignancies. Which treatment should not be prescribed for this patient? A. UVA phototherapy. B. kinesiotherapy. C. vibration massage. D. photodynamic therapy. E. radon-sulfur bath.	C	D	4
19. Patients with lower limb ischemia due to atherosclerosis are recommended to: 1) exercises such as squats, stair climbing, and standing on tiptoes; 2) Buerger's vascular exercises; 3) walking training, preferably three times a day, with exertion close to the onset of pain; 4) limb exercises in water, preferably mineral water, at a temperature of 28-30°C; 5) interval training on a cycle ergometer once a day, 6 days a week. The correct answer is: A. all of the above. B. 1, 3, 4. C. 1, 2, 5. D. 2, 5. E. 2, 4, 5.	A	B	4
20. A 64-year-old man was referred to a spa hospital after radical surgery for left kidney cancer four years ago. Follow-up examinations six months ago revealed no recurrence. The patient has a certificate from his oncologist confirming there are no contraindications to a spa stay. What should be done in this case? A. Admit the patient for treatment, but exclude balneological treatments. B. Admit the patient for treatment, but exclude physical treatments. C. Admit the patient for treatment and administer all types of treatments. D. Admit the patient, but do not administer any treatments. E. Disqualify the patient from spa treatment and send the patient home.	E	C	5
21. Indicate the incorrect method of combining physiotherapy with pharmacotherapy (type III): A. Interferential current and a drug that disintegrates into ions in an aqueous solution. B. Galvanic current and a drug that disintegrates into ions in an aqueous solution. C. Ultrasound and a drug in gel or ointment form. D. Radiant energy and a photosensitizing drug. E. Ultraviolet radiation and a photosensitizing drug.	A	A	5
22. Basic therapeutic massage techniques include: 1) stroking; 2) tapping; 3) rubbing; 4) kneading; 5) vibration. The correct answer is: A. 2, 4, 5. B. 1, 3, 4. C. 1, 2, 4. D. 2, 3, 5. E. all of the above.	E	E	5
23. A four-chamber bath is a bath: 1) using alternating current; 2) which, in terms of galvanic current, is equivalent to a full electric-water bath; 3) using domestic water at a temperature of 38°C; 4) using direct current; 5) in which the direction of current flow can be changed during the treatment. The correct answer is: A. only 1. B. 1.5. C. 3.4. D. 2.3.4. E. 1.2.3.	D	B	4
24. The following are not contraindications to nitrogen cryotherapy in a cryochamber: A. epilepsy. B. pregnancy. C. psoriatic arthritis. D. Raynaud's disease. E. open wounds and ulcers.	C	C	5
25. W laseroterapii zaleca się nieprzekraczanie w trakcie jednego zabiegu dawki: A. 50 J. B. 200 J. C. 500 J. D. 2000 J. E. 5000 J.	B	C	4
26. Identify the false statement regarding the use of ultrasound treatments: A. Ultrasound treatment of small joints (hands, feet) can be performed in a water bath. B. If pain occurs or worsens during ultrasound treatment, the treatment should be discontinued. C. Before ultrasound treatment, it is recommended to use other treatments designed to reduce muscle tension (e.g., Sollux, paraffin wraps). D. Direct ultrasound treatment of blood vessels is contraindicated. E. Direct ultrasound treatment of peripheral nerves is contraindicated.	E	C	4
27. Magnetotherapy does not use the following field shape changes: A. rectangular. B. triangular. C. elliptical. D. semi-rectangular. E. sinusoidal.	C	C	4
28. Indicate the incorrect electrode placement in Träbert's segmental therapy: A. thoracolumbar. B. cervical. C. lumbosacral. D. cervicothoracic. E. upper thoracic.	D	D	4
29. The following are not contraindications to spa treatment: A. Pseudarthrosis following a tibial fracture. B. Rectovaginal fistula. C. An episode of paroxysmal atrial fibrillation occurred 9 months ago. D. Kidney cancer following successful radical resection 2 years ago. E. Pregnancy in the first trimester.	C	A	4
30.Spa treatment facilities do not include: A. a landscaped section of the seashore. B. a graduation tower. C. a spa rehabilitation pool. D. a spa clinic. E. a spa pump room.	D	A	5
31. Inhalation medications include: 1) anticholinergics; 2) mucolytics; 3) glucocorticosteroids; 4) β2-agonists; 5) cholinergics. The correct answer is: A. 1, 4, 5. B. 2, 3, 4, 5. C. 2, 3. D. 1, 2, 3, 4. E. 2, 3, 4.	D	D	5
32. To prolong remission of gastric and duodenal ulcers, beneficial effects of crenotherapy have been found in the use of waters: A. sulfur-hydrogen, sulfur, and sodium chloride. B. bicarbonate and radon. C. thermal and alkaline. D. alkaline and magnesium chloride. E. sodium chloride and bicarbonate.	E	X	X
33. To eliminate excessive and intense balneological reactions, it is necessary to: 1) avoid the full range of therapeutic procedures for 3-4 days; 2) conduct intensive patient monitoring during the first week of treatment; 3) discontinue all stimulating procedures for 3-4 days; 4) follow the indications and contraindications for spa treatment; 5) limit physical exertion for 3-4 days. The correct answer is: A. only 3. B. 1, 2, 4, 5. C. 3, 4, 5. D. 2, 3, 4. E. 2, 3, 5.	B	B	4
34. Which treatment option is not available to patients referred to seaside resorts (Świnoujście, Kamień Pomorski, Kołobrzeg, Dąbki, Ustka, and Sopot)? A. Gynecological diseases. B. Lower respiratory tract diseases. C. Rheumatological diseases. D. Orthopedic and traumatic diseases. E. Cardiological diseases and hypertension.	A	A	4
35. Symptoms of a spa reaction include: 1) anemia; 2) headaches; 3) sleep disturbances; 4) orthostatic syncope; 5) leukocytosis. The correct answer is: A. 1, 3, 4. B. 2, 3, 5. C. 1, 4, 5. D. 3, 4, 5. E. only 2.	B	B	4
36 . Indications for wet carbonic acid baths include: 1) osteoarthritis; 2) compensated coronary artery disease; 3) hypertension; 4) psoriasis; 5) compensated heart disease. The correct answer is: A. 1, 3. B. 1, 3, 5. C. 2, 3, 5. D. 3, 4, 5. E. only 5.	C	B	4
37. The following are not abioliths used in spa peloidotherapy treatments: A. peat. B. medicinal clay. C. loess. D. gyttja. E. fango.	A	C	5
38. For patients with chronic hypoacid gastritis, drinking water should be used: A. radon water. B. ferrous acidic water. C. alkaline acidic water. D. sulphate water. E. sodium chloride water (up to 1.5%).	E	E	4
39. Indications for ozone therapy with a mixture of oxygen and ozone include: 1) viral keratitis; 2) diabetic foot; 3) pressure ulcer; 4) vitamin E deficiency; 5) respiratory diseases. The correct answer is: A. 2,3. B. 1,4,5. C. 4,5. D. 1,2,3. E. 2,3,5.	D	D	4
40. To reduce bronchial swelling during mineral water inhalation, use: A. salt water containing 0.8% NaCl. B. salt water containing 1.5% NaCl. C. bicarbonate-containing water, rs containing Ca. D. radium-containing waters. E. waters containing siarczany.	C	B	4
41. The following are not included in the separate treatment profiles of Polish spas: A. diabetes. B. lower respiratory tract diseases. C. upper respiratory tract diseases. D. ophthalmological diseases. E. pediatric diseases.	E	D	5
42. Contraindications to cancer treatment within 5 years of completing treatment include the following cancers: 1) breast cancer; 2) kidney cancer; 3) malignant melanoma; 4) laryngeal cancer; 5) malignant lymphoma. The correct answer is: A. 2, 3, 5. B. 1, 4. C. 3, 4, 5. D. 1, 2, 4. E. 3, 5.	A	A	4
43 .The following electrotherapeutic procedures should be used in a patient with flaccid (peripheral) brachial plexus paresis: 1) anodic galvanization; 2) cathodic galvanization; 3) triangular pulse electrostimulation; 4) tonolysis; 5) rectangular pulse electrostimulation. The correct answer is: A. 2, 3, 5. B. 2, 3. C. 1, 4. D. 1, 3, 5. E. only 3	B	B	4
44.Parametry do zabiegu impulsowym polem niskiej częstotliwości u pacjenta leczonego w uzdrowisku z powodu zaawansowanej choroby zwyrodnieniowej stawów biodrowych powinny wynosić: A. częstotliwość 0,1-0,5 Hz, indukcja poniżej 3 mT. B. częstotliwość 1-5 Hz, indukcja poniżej 5 mT. C. częstotliwość 10-50 Hz, indukcja poniżej 5 mT. D. częstotliwość 20-50 Hz, indukcja powyżej 5 mT. E. częstotliwość 50-100 Hz , indukcja powyżej 5 mT.	D	C	4
4 5. For phonophoresis, a patient with degenerative changes in the knee joints should receive ultrasound at the following frequencies: A. 1 MHz and lignocaine gel. B. 1 MHz aa nd nonsteroidal anti-inflammatory drug gel. C. 3 MHz and nonsteroidal anti-inflammatory drug gel. D. 3 MHz and heparin gel. E. 3 MHz and lignocaine gel.	B	B	5
46. A blue filter during Sollux lamp therapy is useful for patients with: A. trigeminal neuralgia. B. as preparation for a massage treatment. C. peripheral paresis of the median nerve in the 4th month of the disease. D. acute inflammatory skin condition - erythroderma. E. skin eruptions associated with herpes zoster.	A	A	5
47. The main mechanism of body overheating during an infrared sauna session is: A. conduction. B. convection. C. convection. D. conduction. E. radiation.	E	E	5
48.​​ In patients with painful shoulder syndrome, cryostimulation treatments such as: 1) cold packs; 2) cold air cryotherapy; 3) carbon dioxide cryotherapy; 4) nitrogen vapor cryotherapy; 5) cold compresses are beneficial to reduce pain and prepare for exercise. The correct answer is: A. 1.5. B. 1.4. C. 2.3.4. D. 3.4. E. only 4.	C	C	5
49. The Scottish douche uses the following therapeutic stimuli: 1) water at a temperature of 10-15°C for approximately 1-2 minutes; 2) water at a temperature of 38-42°C for several seconds; 3) water at a temperature of 38-42°C for approximately 1-2 minutes; 4) water at a temperature of 10-15°C for several seconds; 5) water at a neutral temperature and variable pressure. The correct answer is: A. only 5. B. 3.4. C. 1.3. D. 1.2. E. 3.5.	B	B	5
50. Terrain therapy should not be used in patients with: 1) hypertension in stages III and IV; 2) neurosis; 3) obesity; 4) acute respiratory disease; 5) degenerative hip disease. The correct answer is: A. 1, 4. B. 1, 2, 4. C. 1, 3, 4. D. 3, 4, 5. E. 3, 5.	A	A	5
51. The rubbing technique used in classical massage is intended to affect: 1) joint capsules; 2) tendons; 3) fascia; 4) veins and lymphatic vessels; 5) muscles. The correct answer is: A. 3, 4, 5. B. 2, 3, 5. C. 1, 2, 3. D. 1, 3, 5. E. only 5.	C	B	5
52. A patient undergoing surgery at a spa for a pertrochanteric femur fracture should be prescribed the following: 1) a brine bath; 2) low-frequency magnetic therapy of the pelvic girdle; 3) calcium iontophoresis of the hip joint; 4) kinesiotherapy; 5) mud iontophoresis of the femoral trochanter area. The correct answer is: A. 1,2,4. B. 2,4. C. 1,2,3. D. 1,3,4. E. 1,4,5.	A	A	5
53. The temperature of the mud used as a vaginal tampon in a patient undergoing treatment at a spa for chronic ovarian inflammation should be: A. 35-38°C. B. 38-40°C. C. 40-42°C. D. 42-45°C. E. 46-50°C.	D	B	4
54. In the balneological treatment of skin lesions in patients with psoriasis, beneficial therapeutic effects can be achieved by combining: A. IR irradiation with a red filter before iodine iontophoresis. B. UV irradiation before calcium iontophoresis. C. Paraffin treatments and kinesiotherapy. D. General cryotherapy with kinesiotherapy. E. Brine baths and UV irradiation.	E	E	5
5 5.PUVA therapy is the use of: A. UVB rays. B. UVA rays. C. UVA rays combined with medications (psoralens). D. the entire ultraviolet spectrum. E. a portion of the ultraviolet spectrum.	C	C	5
56. Photosensitization is: A. increased skin sensitivity to ultraviolet radiation. B. skin insensitivity to ionizing radiation. C. lack of skin response to physical factors. D. abnormal bathing reaction. E. skin insensitivity to infrared radiation.	A	A	5
57. The characteristics of laser radiation are: A. diffraction, dispersion, and diffraction. B. monochromaticity, coherence, collimation, and intensity. C. coherence and diffraction. D. penetration into all skin layers. E. diffraction and absorbance.	B	B	5
58. In laser therapy for acute conditions, the following doses are used: A. 0.1-3 J/cm². B. 3-6 J/cm². C. 6-9 J/cm². D. 9-12 J/cm². E. 9-14 J/cm².	A	A	5
59. Indications for laser biostimulation include: A. difficult-to-heal wounds. B. osteoarthritis. C. acne vulgaris. D. neuropathies. E. all of the above.	E	E	5
60. The dosage of ultraviolet radiation depends on: A. the intensity of the emitted radiation. B. the patient's individual sensitivity. C. the distance of the light source from the patient's body. D. the exposure time. E. all of the above.	E	E	5
61. The generation of ultrasound is associated with: A. Lorenz force. B. Eddy current. C. Lewis wave. D. Piezoelectric effect. E. Wien's law.	D	D	5
62. ERA - the active surface area of ​​the ultrasonic head is: A. 10-20% of the actual surface area of ​​the ultrasonic head. B. 30-40% of the actual surface area of ​​the ultrasonic head. C. 50-60% of the actual surface area of ​​the ultrasonic head. D. 85-95% of the actual surface area of ​​the ultrasonic head. E. 100% of the actual surface area of ​​the ultrasonic head.	D	D	5
63. Treatments using pulsed ultrasound waves are intended to: A. increase micromassage and microfluid flow in the interstitial fluid. B. Reduce the thermal effect. C. increase the thermal effect. D. induce stable cavitation. E. reduce the micromassage.	B	B	5
64. The motor point of a completely denervated muscle undergoes: A. complete atrophy. B. proximal shift. C. distal shift. D. more superficial shift. E. unchanged.	A	C	4
65. A quantitative assessment of nerve function is not: A. degenerative reaction. B. curve. C. chronaxie. D. rheobase. E. accommodation quotient.	A	A	5
66. An accommodation coefficient of 1 indicates: A. partial denervation of the muscle. B. vegetative neurosis. C. no damage. D. increased muscle adaptability to slowly increasing current intensity. E. complete muscle degeneration.	E	E	5
67. When a neurogenically damaged muscle regenerates, the coefficient of accommodation: A. decreases. B. does not change. C. increases. D. first increases, then decreases. E. first decreases, then increases.	C	C	5
68. For peripheral nerve palsy, the following treatments are recommended: A. laser, anodic galvanization, and electrical stimulation. B. infrared radiation, ascending galvanization, electrical stimulation. C. cryotherapy, cathodic galvanization. D. paraffin, descending galvanization. E. cryotherapy, low-frequency alternating magnetic field.	B	A	5
69. A 74-year-old woman with a history of stroke and spastic right-sided paralysis and hypertension (average blood pressure 170/90) was referred to a spa hospital in Ciechocinek. Physical therapy included: A. laser therapy, kinesiotherapy. B. carbonic acid baths, tonolysis, low-frequency alternating magnetic field, kinesiotherapy, and massage. C. low-frequency alternating magnetic field, massage. D. underwater massage, kinesiotherapy. E. kinesiotherapy.	B	E	5
70. A 75-year-old patient with osteoarthritis and an implanted pacemaker is being treated at the spa hospital in Busko-Zdrój. The following physical therapy procedures should be recommended: A. cryotherapy, kinesiotherapy. B. cryotherapy, ultrasound. C. sulfur-hydrogen sulfur baths, cryotherapy, kinesiotherapy, and massage. D. diadynamic currents, kinesiotherapy. E. kinesiotherapy, massage.	C	A	5
71. A 73-year-old patient with low back pain is being treated at a spa hospital in Ciechocinek. The following physical therapy should be recommended: A. diadynamic currents, kinesiotherapy. B. Nemec currents 50-100 Hz, laser therapy, kinesiotherapy,and massage. C. cryotherapy, massage. D. kinesiotherapy, massage. E. brine baths, kinesiotherapy.	B	B	4
72. A property of solar radiation is its ability to activate the production of vitamin D in the skin. The following radiation is responsible for this process: A. UV-B. B. UV-A. C. UV-C. D. visible light. E. infrared.	A	A	5
73. Contraindications to sauna treatments include: 1) coronary artery disease and conditions following a heart attack; 2) exacerbation of rheumatic disease; 3) cerebral atherosclerosis; 4) susceptibility to infections and colds; 5) thyroid disease. The correct answer is: A. 2,3,4. B. 1,2,3. C. 1,3,5. D. 1,2,3,5. E. 1,2,4,5.	D	B	5
74 . The hydrostatic effect of water causes: A. a decrease in intracardiac pressure. B. a decrease in abdominal and chest circumference. C. a decrease in vascular resistance. D. a decrease in right ventricular systolic pressure. E. a decrease in venous blood outflow.	B	B	5
75. The effects of laser radiation: 1) are related to the Arndt-Schultz law; 2) result from secondary reactions; 3) reduce phagocytosis; 4) at low doses, it has a stimulating effect, stimulating biological activity in tissues; 5) strong stimuli have an inhibitory effect. The correct answer is: A. 1,2,3. B. 1,3,4. C. 2,3,5. D. 1,2,4,5. E. 3,4,5.	D	D	5
76. Magnetostimulation is a therapy using low-induction magnetic fields. Its properties do not include: A. the frequency of the magnetic wave envelope is lower than the fundamental frequency of the application. B. The limitation of electromagnetic wave penetration by water and organic structures. C. the biological effect associated with ion cyclotron resonance. D. the ability to emit a uniform magnetic field through therapy applicators. E. the ability to combine magnetostimulation with light therapy in the form of magneto-LED therapy.	B	B	5
77. The therapeutic effect of ultrasonic waves is primarily related to: A. their influence on the ability to diffuse through cell membranes. B. the cavitation effect. C. increased temperature of the sonicated tissues. D. mechanical action. E. the formation of free radicals.	D	C	5
78. Clinical considerations for rehabilitation are defined as both physician-dependent physician-independent. These include: 1) location of the injury; 2) method of injury management; 3) secondary extent of the injury; 4) fracture management; 5) fracture management. The correct answer is: A. only 1. B. 2, 4, 5. C. 2, 3, 4, 5. D. only 2. E. 3, 4, 5.	B	B	5
79. Bioliths include: A. silts. B. gyttjas. C. marine sediments. D. lake sediments. E. spring sediments.	A	B	4
80. Aerosol types have been identified according to the Łaukajtys and Madeyski classification. Which answer correctly describes the type: A. microparticle containing approximately 50% microparticles? B. fine-particle containing approximately 50% fine particles? C. medium-particle containing approximately 50% medium particles? D. coarse-particle containing approximately 50% large particles? E. heterogeneous particle containing particles of various sizes, but 50% of which are not of any size?	E	E	4
81 . A patient with Raynaud's syndrome will benefit most from: 1) transcutaneous electrical nerve stimulation (TENS); 2) cryochamber; 3) radon baths; 4) dry carbonic acid baths; 5) biostimulation laser. The correct answer is: A. 1, 2. B. 3, 4. C. 1, 5. D. 2, 5. E. 2, 3.	B	B	4
82. Contraindications to the use of shortwave diathermy include: 1) implanted cardiac pacemaker; 2) chronic otitis media; 3) menstruation; 4) chronic neuralgia; 5) presence of metal in the body. The correct answer is: A. 1, 3, 5. B. 2, 4. C. 1, 2, 4. D. 4, 5. E. 1, 4.	A	A	5
83. Natural climatotherapy methods do not include: A. heliotherapy. B. thalassotherapy. C. halotherapy. D. aerotherapy. E. terrain therapy.	C	C	5
84 . In spa treatment of psoriasis, the most beneficial therapeutic effects are achieved by: 1) sulfur-hydrogen sulfur baths; 2) segmental massage; 3) radon baths; 4) PUVA; 5) halotherapy. The correct answer is: A. 1,3,5. B. 1,4,5. C. 2,4,5. D. 3,4,5. E. 1,3,4.	E	E	5
85. Immediately after a local cryotherapy treatment, the patient should be prescribed: A. laser therapy. B. magnetic stimulation. C. exercise. D. rest, preferably in a lying position. E. segmental massage.	C	C	5
86. A 70-year-old patient referred for spa treatment with stable coronary artery disease, a history of myocardial infarction and coronary angioplasty with BMS stent implantation two years earlier, and thoracic spine pain due to degenerative changes should not be prescribed: A. classical massage. B. dry carbonic acid baths. C. individual exercise classes. D. polarized light. E. phonophoresis with diclofenac paraspinally on the thoracic spine.	E	B	4
87. In patients with lead poisoning, drinking the following will be effective: A. Bicarbonate waters. B. Sulfur-hydrogen sulfur waters. C. Sodium chloride waters. D. Arsenic waters. E. Ferrous waters.	B	B	4
88. For a patient with muscle atrophy resulting from prolonged immobilization due to a femur fracture, the following therapy should be used: A. diadynamic current (DF). B. interferential currents with a frequency of 0-100 Hz. C. ultrasound. D. Kotz currents. E. transcutaneous electrical nerve stimulation (TENS).	D	D	5
89. A 66-year-old patient was treated on an outpatient basis in the afternoon for chronic right-sided sciatica due to a herniated disc at L5-S1. He has been experiencing insomnia since completing a physiotherapy program. Which treatment could be causing the patient's insomnia? A. magnetotherapy. B. laser therapy. C. McKenzie's method of gymnastics. D. interferential currents. E. segmental massage.	A	C	4
90. Brine is sodium chloride water containing at least: A. 0.5% sodium chloride. B. 1.6% sodium chloride. C. 2% sodium chloride. D. 5% sodium chloride. E. 10% sodium chloride.	B	B	5
91. The recommended parameters for magnetic therapy used for osteoarthritis are: A. Rectangular pulse waveform, daily treatments, 8-10 mT induction. B. Rectangular pulse waveform, treatments every three days, 25 mT induction. C. Triangular pulse waveform, daily treatments, 15 mT induction. D. Triangular pulse waveform, treatments every two days, 5 mT induction. E. Sinusoidal pulse waveform, daily treatments, 2 mT induction.	A	A	4
92. A 38-year-old patient, who was undergoing knee surgery following a bicycle injury (surgery performed 4 months ago), consulted her physician on the 12th day of her stay due to worsening pain in the operated knee joint and minor swelling that had been present for two days. She is also concerned about general weakness. Which statement is true? A. The patient has bacterial inflammation of the knee joint after surgery, and antibiotics should be administered, and the procedures discontinued for 5 days. B. The patient's treatment regimen is poorly chosen and should be changed. C. The patient likely did not complete all the procedures, which is why the condition worsened. D. The patient has new inflammation of the operated knee joint, and cryotherapy should be administered to the knee area. E. The patient is experiencing a spa-like reaction. The current treatment regimen should be continued.	E	E	4
93. The characteristics of a full brine bath in a bathtub are: A. Water temperature: 25-27°C, sodium chloride concentration: 3.5-4.0%, bath duration: 10 minutes, treatment daily. B. Water temperature: 33-34°C, sodium chloride concentration: up to 5.5%, bath duration: 10-12 minutes, treatment every 3 days. C. Water temperature: 36-37°C, sodium chloride concentration: 3-4%, bath duration: 15-20 minutes, treatment 3 times a week, every other day. D. Water temperature: 40-42°C, sodium chloride concentration: 6-7%, bath duration: 15 minutes, treatment on Mondays and Thursdays. E. Water temperature: 43-44°C, sodium chloride concentration: 0.5-1.0%, bath duration: 20 minutes, treatment daily.	C	B	4
94. The parameters of peloid therapy used for vaginal tampons (columnization) for gynecological conditions are: A. Natural peloid at a temperature of 34-37°C, treatment time: 14 minutes, every 2 days. B. Crushed peloid at a temperature of 31-32°C, treatment time: 10-20 minutes, every 3 days. C. Sterile peloid suspension at a temperature of 45-48°C, treatment time: 5 minutes, daily. D. Peloid paste at a temperature of 27-29°C, treatment time: 25 minutes, 4 times a week. E. Sterile peloid paste at a temperature of 42-45°C, treatment time: 15-20 minutes, 3-6 times a week.	E	E	5
95. A 54-year-old woman with type 2 diabetes (she has had it for 2 years), a BMI of 33 kg/m², and WHO stage 1 hypertension was referred to Polanica-Zdrój. Indicate the appropriate balneophysical procedure: 1) standard crenotherapy with medium water (Wielka Pieniawa); 2) impact crenotherapy with water (Krystynka); 3) brine bath, sodium chloride concentration 0.5-1.0%, temperature 28°C; 4) group exercise in a pool or indoor gym; 5) pearl bath with hydromassage every other day. The correct answer is: A. 1,2,4. B. 2,3,5. C. 2,5. D. 1,3,5. E. 1,4,5.	E	E	4
96. A 5-year-old girl living in Katowice frequently suffers from upper respiratory tract infections. She has been attending preschool for two years. Four months ago, she had her adenoids removed. Which spa should the child be referred to? A. Busko-Zdrój. B. Połczyn-Zdrój. C. Długopole-Zdrój. D. Dąbki. E. Nałęczów.	D	D	4
97. A 67-year-old patient who has suffered from spinal degeneration and type 2 diabetes for 10 years underwent surgery seven months ago to remove a 4 mm diameter squamous cell carcinoma from his forehead. He received a National Health Fund (NFZ) referral to the Krynica Zdrój spa. He leaves in 10 days. Which statement is true? A. The patient is in dire need of rehabilitation and can now go to a health resort for treatment using saline water inhalation and spinal peloidotherapy. B. Due to his age, the patient should receive a referral to the Nałęczów spa. C. During his stay at the health resort, the patient can only benefit from kinesiotherapy and thalassotherapy. D. The patient cannot go to a health resort until 12 months after the squamous cell carcinoma removal procedure. E. The patient can go to a health resort and has no contraindications to treatment.	D	D	5
98. Contraindications to systemic UV-A (PUVA) exposure include: 1) rheumatoid arthritis during gold therapy; 2) psoriasis vulgaris; 3) pulmonary tuberculosis; 4) vitiligo; 5) cataracts and glaucoma. The correct answer is: A. 1, 3, 5. B. 1, 4, 5. C. 2, 3, 4. D. 1, 3, 4. E. 4, 5.	A	A	5
99. What is chronaxie? A. The minimum current intensity that induces a muscle contraction using a rectangular pulse with a duration of 500 ms. B. The minimum current intensity is obtained using triangular pulses with a duration of 100 ms. C. The angle of inclination relative to the time axis at which a contraction can be achieved. D. The shortest duration of an electrical pulse, expressed in milliseconds (ms), that induces a contraction of the tested muscle using a current intensity equal to twice the rheobase. E. The minimum current intensity is achieved using mixed pulses with a duration of 2500 ms.	D	D	5
100. Indications for the use of ultrasound include: 1) pain syndrome associated with degenerative joint and spine disease; 2) ossification disorders associated with rickets – procedures on the epiphyses of bones in children over 8 years of age; 3) inflammation and thrombosis of the venous vessels of the upper limbs; 4) trophic changes and tendon contractures (e.g., Dupuytren's contracture); 5) gout. The correct answer is: A. 1, 4, 5. B. 2, 3, 4, 5. C. 1, 5. D. 1, 2, 3. E. 3, 4, 5.	A	A	4
101. A 74-year-old woman with type 2 diabetes, hypertension, and leg ulcers due to venous insufficiency was referred for laser therapy. The recommended laser therapy parameters for the patient were: A. soft helium-neon laser, daily, 5-12 J/cm², 10-20 minutes. B. hard laser, once a week, 1-3 J/cm², 25-30 minutes. C. high-energy ablative laser (Er;YAG), 7 J/cm², twice a week, 15 minutes. D. biostimulating laser, three times a week, 14-20 J/cm², 15 minutes. E. fractional non-ablative semiconductor diode laser, daily, 20-25 J/cm², 5 minutes.	A	A	4
102. Indications for low-temperature sauna treatments include: A. biological regeneration, tuberculosis, chronic gastritis, and kidney stones. B. biological regeneration, sleep disorders, spinal degeneration, neuralgia. C. sensory disturbances, anorexia, body care, subacute appendicitis. D. psoriasis, impaired blood circulation in the lower limbs, difficult-to-heal wounds, and neurodermatitis. E. fevers up to 38-39°C, skin care, sleep disorders, and sensory disturbances.	B	B	4
103. Indications for local cryotherapy treatments using liquid nitrogen include: 1) inflammatory joint changes associated with gout; 2) advanced atherosclerosis with trophic changes; 3) swelling around the ankle joint two days after injury; 4) Sudeck's syndrome; 5) fibromyalgia. The correct answer is: A. 2,3,4. B. 1,3,5. C. 1,3. D. 1,2,4. E. 2,4,5.	B	B	4
104. A 52-year-old patient with coronary heart disease, 5 months post-infarction, BMI of 27 kg/m², WHO stage I hypertension, and smoking cessation. The planned Borg stress level is 10-12 (light to moderate stress). The recommended kinesiotherapy treatments for this patient are: A. passive. B. aerobic exercises combined with breathing exercises. C. Kegel exercises. D. antigravity and elongation exercises. E. Vojta method.	B	B	5
105. "One form of neurorehabilitation, focused on working with children with cerebral palsy, involves muscle stimulation through various motor exercises, which helps activate the patient's brain, inhibits abnormal reflexes, and triggers reflexes that are as close to normal as possible." This is a description of a special method used in kinesiotherapy called: A. Fascial Distortion Model (FDM) method. B. Haas method. C. Bobath method. D. Dorn method. E. Mennell method.	C	C	5
106. A 37-year-old patient was referred to Ciechocinek for rehabilitation following a massive knee injury, a bicycle accident (arthroscopy of the right joint 9 months ago). She is currently pain-free. For several years, the patient has also been treated for lymphedema of the lower legs and feet, with venous insufficiency. According to the patient, these are her main complaints. Her BMI is 28 kg/m². Of the balneophysical treatment methods listed below, the following program should be used: A. brine bath, mud wrapping at 45-46°C for the lower limbs, magnetic therapy for the lower legs, and kinesiotherapy. B. brine bath, L-S spine cryotherapy, Sollux lamp for the ankle joints, whirlpool bath for the lower limbs. C. Carbon dioxide gas bath, magnetic therapy for the knee joints, manual lymphatic drainage with pneumatic massage, pool exercises, whirlpool bath for the lower limbs. D. Radon bath, mud wraps for the calves, magnetic therapy, kinesiotherapy. E. Radon bath, whole-body cryotherapy, mud wraps for the lower limbs, shortwave induction diathermy for the knee joints.	C	C	5
107. An 87-year-old woman was referred to Inowrocław. The patient suffers from knee osteoarthritis, with a right knee endoprosthesis implanted in 2022, and she moves using a walker. She also has osteoarthritis of the small joints of the hands with significant deformities and grade I hypertension (WHO). The patient is awaiting surgery for cataracts in both eyes and arrived with a caregiver. In balneophysiotherapy treatment, which of the following should be applied? Options: A. Radon bath 28–34°C, gymnastics in a brine pool, peat paste compresses on the wrist joints, interferential currents on the knee joints. B. Carbonic acid bath, peat paste compresses on the hands, Dorn method gymnastics, electrostimulation of both lower limbs, whirlpool bath for lower limbs. C. Carbonic acid gas bath, magnetotherapy of knee joints, brine whirlpool bath for upper limbs, peat compresses on the hands, and individual active-passive gymnastics. D. Brine bath 44–46°C, gymnastics in the pool, laser therapy on wrist joints alternating with ultrasound, peat paste compresses on the knees, ultrasound on the knee joints. E. Brine bath 38–40°C, individual passive gymnastics, peat compresses on the hands, laser therapy on wrist joints, ultrasound on the knees with anti-inflammatory medication.	C	C	5
108. A 10-year-old child with chronic upper respiratory tract inflammation, a fracture of the distal part of the right radius near the wrist (3 months ago), and obesity has been referred to the Kołobrzeg spa. The child is very nervous and has poor sleep. Question: Indicate the procedures contraindicated for this child: 1 Inhalations and seaside walks 2 Radon bath 3 Group gymnastics in a brine pool 4 Ultrasound of the right wrist area with peat paste 5 Shortwave diathermy on the area of the fractured right radius Answer options: A. 1, 3, 4 B. 1, 2, 4 C. 2, 4, 5 D. 1, 2, 5 E. 2, 3, 5	C	C	5
109. An example of combining balneological and physical therapy treatments of type III is the use of: A. iontophoresis. B. magnetolaser therapy. C. mud treatments and massage. D. carbonic acid baths and vascular exercises. E. cryotherapy and kinesiotherapy.	A	B	5
110. In basic procedures used in the recommended balneotherapy and physiotherapy program to be performed in spa conditions for patients with diabetic foot syndrome with ulceration, which of the following is not included: A. Ozone therapy, B. Magnetotherapy, C. Laser therapy, D. Use of ultrasound, E. Carbonic acid gas bath	D	A	5
111. Periosteal massage is not contraindicated in: A. bone tuberculosis. B. osteoporosis. C. osteomalacia. D. rheumatoid arthritis. E. joint degeneration.	E	E	5
112. The following are not contraindications to the use of full electro-water baths: A. thrombophlebitis. B. nervous hyperexcitability. C. sensory disturbances. D. circulatory failure. E. bleeding diathesis.	B	B	5
113. A physical property characteristic of laser radiation is not: A. monochromaticity. B. collimation. C. coherence. D. linear polarization. E. intensity	D	E	5
114. During color therapy treatments, light of the following colors has a regenerative, slightly stimulating, and antispasmodic effect: A. red. B. orange. C. yellow. D. green. E. blue.	B	B	5
115. The dose rates used in ultrasound therapy procedures are: A. 0.01-0.05 W/cm². B. 0.05-0.5 W/cm². C. 0.5-1.5 W/cm². D. 1.5-2.0 W/cm². E. 2.0-3.0 W/cm².	D	E	5
116. General contraindications to spa treatment do not include: A. severe personality or behavioral disorders. B. debilitating systemic disease. C. hernia prone to incarceration. D. systolic dysfunction below 35%. E. stable coronary artery disease, stage II according to the CCS.	E	E	5
117. Treatment of women's diseases is a medical discipline offered at the following spas: A. Szczawno-Zdrój. B. Kudowa-Zdrój. C. Jedlina-Zdrój. D. Busko-Zdrój. E. Świeradów-Zdrój.	E	A	4
118. The following are not indications for drinking bicarbonate water: A. Chronic prostatitis. B. Chronic gastritis with hypoacidity. C. Ulcerative colitis. D. Diabetes associated with obesity. E. Chronic toxic liver damage.	C	A	5
119. The following are not contraindications to peat treatments: A. hyperthyroidism. B. chronic hepatitis. C. endometriosis. D. anemia. E. hypertension.	B	D	5
120. The therapeutic mechanisms of balneological treatments do not include: A. stimulation of the secretion of prostaglandins PGE2 and LTB4. B. stimulation of endorphin secretion. C. stimulation of the antioxidant system. D. inhibition of the secretion of proinflammatory cytokines. E. increased phagocytosis.	A	A	5

## References

[1] Wang K (2024). From ELIZA to ChatGPT: a brief history of chatbots and their evolution. Appl Comput Eng.

[2] Vaswani A, Shazeer N, Parmar N (2017). Attention is all you need [PREPRINT]. arXiv.

[3] Jin Q, Chen F, Zhou Y (2024). Hidden flaws behind expert-level accuracy of multimodal GPT-4 vision in medicine. NPJ Digit Med.

[4] (2025). GPT-5 surpasses doctors in medical reasoning benchmarks. https://www.ainews.com/p/gpt-5-surpasses-doctors-in-medical-reasoning-benchmarks?utm_source=chatgpt.com.

[5] Tikhomirov L, Semmler C, McCradden M, Searston R, Ghassemi M, Oakden-Rayner L (2024). Medical artificial intelligence for clinicians: the lost cognitive perspective. Lancet Digit Health.

[6] Protano C, Vitali M, De Giorgi A, Marotta D, Crucianelli S, Fontana M (2024). Balneotherapy using thermal mineral water baths and dermatological diseases: a systematic review. Int J Biometeorol.

[7] Protano C, Fontana M, De Giorgi A (2023). Balneotherapy for osteoarthritis: a systematic review. Rheumatol Int.

[8] Koroglu S, Yıldız M (2024). Effectiveness of hydrotherapy and balneotherapy for anxiety and depression symptoms: a meta-analysis. Curr Psychol.

[9] Hsieh CH, Hsieh HY, Lin HP (2024). Evaluating the performance of ChatGPT-3.5 and ChatGPT-4 on the Taiwan plastic surgery board examination. Heliyon.

[10] Kufel J, Paszkiewicz I, Bielówka M (2023). Will ChatGPT pass the Polish specialty exam in radiology and diagnostic imaging? Insights into strengths and limitations. Pol J Radiol.

[11] Yudovich MS, Makarova E, Hague CM, Raman JD (2024). Performance of GPT-3.5 and GPT-4 on standardized urology knowledge assessment items in the United States: a descriptive study. J Educ Eval Health Prof.

[12] Goodings AJ, Kajitani S, Chhor A (2024). Assessment of ChatGPT-4 in family medicine board examinations using advanced ai learning and analytical methods: observational study. JMIR Med Educ.

[13] Lewandowski M, Łukowicz P, Świetlik D, Barańska-Rybak W (2024). ChatGPT-3.5 and ChatGPT-4 dermatological knowledge level based on the Specialty Certificate Examination in Dermatology. Clin Exp Dermatol.

[14] Singhal K, Azizi S, Tu T (2023). Large language models encode clinical knowledge. Nature.

[15] Wójcik S, Rulkiewicz A, Pruszczyk P, Lisik W, Poboży M, Domienik-Karłowicz J (2024). Reshaping medical education: performance of ChatGPT on a PES medical examination. Cardiol J.

[16] Raza MM, Venkatesh KP, Kvedar JC (2024). Generative AI and large language models in health care: pathways to implementation. NPJ Digit Med.

[17] Kufel J, Bielówka M, Rojek M (2024). Assessing ChatGPT's performance in national nuclear medicine specialty examination: an evaluative analysis. Iran J Nucl Med.

